# Acceptability and preliminary effects of the volunteer-supported Meaningful Moments program to engage older adults with advanced dementia on a hospital-based specialized dementia care unit: a mixed methods study

**DOI:** 10.1186/s12877-024-05194-9

**Published:** 2024-07-11

**Authors:** Marie-Lee Yous, Esther Coker, Paulette V. Hunter, Kathryn A. Fisher, Joanna L. Sue, Maria Nicula, Nadia Kazmie, Theresa Orsini, Tamara Sussman, Genevieve Thompson, Sharon Kaasalainen

**Affiliations:** 1 School of Nursing, McMaster University, 1280 Main Street West, Hamilton, ON L8S 4K1 Canada; 2 Department of Psychology, St. Thomas More College, University of Saskatchewan, 1437 College Drive, Saskatoon, SK S7N 0W6 Canada; 3 Department of Psychiatry and Behavioural Neurosciences, McMaster University, 1280 Main Street West, Hamilton, ON L8S 4K1 Canada; 4 Health Research Methodology, McMaster University, 1280 Main Street West, Hamilton, ON L8S 4K1 Canada; 5 School of Social Work, McGill University, 3506 University St, Montreal, QC H3A 2A7 Canada; 6 College of Nursing, University of Manitoba, 89 Curry Place (Fort Garry Campus), Winnipeg, MB R3T 2N2 Canada

**Keywords:** Dementia, Older adults, Family, Engagement, Quality of life, Hospital-based specialized dementia care unit, Post-acute care setting

## Abstract

**Background:**

Namaste Care offers practical skills for healthcare providers, volunteers, and families to meaningfully engage individuals with dementia in activities (e.g., music, massage, reminiscing, socialization, aromatherapy, snacks). A hospital-based specialized dementia care unit for patients with mid- to late-stage dementia offered an adapted version of the Namaste Care program, which was called Meaningful Moments. The aim of this study was to assess the acceptability and preliminary effects of this novel approach using trained volunteers for older adults with mid- to late-stage dementia.

**Methods:**

A mixed methods multiphase design was used. Qualitative description was used to explore acceptability of the Meaningful Moments program delivered over 6 months through focus groups (e.g., charge nurses, therapeutic recreationists, nurses, social workers) and individual interviews with one volunteer and two family members. A prospective pre-post-test study design was used to evaluate the preliminary effects of the program for patients with dementia and family members. Outcomes included quality of life, neuropsychiatric symptoms, and pain for patients with dementia and family carer role stress and the quality of visits for families. Data were collected from June 2018 to April 2019. Descriptive analyses of participants’ characteristics were expressed as a mean (standard deviation [SD]) for continuous variables and count (percent) for categorical variables. Focus group and individual interview data were analyzed using thematic analysis. The generalized estimating equations (GEE) method was used to assess change in the repeated measures outcome data.

**Results:**

A total of 15 patients received the Meaningful Moments interventions. Families, staff, and volunteers perceived that patients experienced benefits from Meaningful Moments. Staff, volunteers, and families felt fulfilled in their role of engaging patients in the Meaningful Moments program. Individualized activities provided by volunteers were perceived as necessary for the patient population. There were no statistically significant improvements in patient outcomes. There was a statistically significant decline in family carer role stress.

**Conclusions:**

Using a one-on-one approach by volunteers, patients experienced perceived benefits such as improved mood and opportunities for social interactions. There is a need for tailored activities for older adults with advanced dementia through practical strategies that can offer benefit to patients.

## Background

Specialized care units in long term care (LTC) homes (i.e., care homes, skilled nursing facilities) and post-acute settings (i.e., areas providing services for patients who are considered too stable for a hospital but not yet ready to be discharged home) have been created since the 1980’s [1] in response to the health and mental healthcare needs of a growing number of older adults living with mid- to late- stage dementia. These settings are also known as memory care or dementia units. At this stage, support needs must be understood holistically, not only through a person’s direct requests, but also through their routines and patterns, and their expressions and behaviours. Staff in specialized care units are trained to recognize actions and expressions that might convey unmet needs, such as being hungry, thirsty, afraid, or bored. The language ‘responsive behaviors’ is sometimes used to acknowledge these changes in self-expression and how they are shaped by addressing underlying needs [2, 3]. Person-centred non-pharmacological interventions such as music therapy and staff training in communication and other person-centred care approaches are safer to use compared to pharmacological approaches and have been found to reduce agitation and anxiety for older adults experiencing responsive behaviours associated with dementia [4]. Staff in specialized care units are therefore encouraged to rely on non-pharmacological strategies before considering pharmacological approaches (e.g., pain medications, antipsychotics, antidepressants) when supporting the psychosocial care of older adults living with dementia.

One person-centred, non-pharmacological psychosocial intervention for people living with advanced dementia is Namaste Care [5]. Namaste care has been shown to have great potential in LTC settings to improve quality of life, dignity, and comfort of individuals in the later stages of dementia [6]. This sensory program is delivered through a combination of activities including music, massage, reminiscing, socialization, aromatherapy, and snacks. Namaste Care offers practical skills for healthcare providers (HCPs), volunteers, and families to meaningfully engage individuals with dementia in activities. Since its creation Namaste Care has been used internationally in multiple settings including LTC, hospice, acute care, and home settings [3, 7–10].

In LTC settings, Namaste Care has led to benefits for persons with dementia such as reduced use of antianxiety medications and other psychotropic medications, lower risk of delirium, decreased pain symptoms, and fewer responsive behaviours while improving quality of life and relationships with staff [9, 11–15]. In acute care settings, Namaste Care has been perceived by HCPs as a way to reduce agitated behaviour, enhance communication between persons with dementia and staff, and improve quality of life for people with dementia [7, 10]. A recent study found that HCPs, family members, and volunteers reported improved mood, less anxiety, and higher levels of alertness in LTC homes where Namaste Care was implemented [9]. The Namaste Care program in LTC has benefited from the engagement of volunteers, who have played an important role in supporting families and building positive relationships [16]. Based on a review of previous studies, for Namaste Care to be successfully implemented, it was important to: (a) work with individual LTC homes to determine how best to implement the program within the context of their own setting; (b) ensure that staff will prioritize the program; and (c) involve volunteers as program facilitators. Considering these factors, the Namaste Care program underwent adaptations to better meet the unique context of the study site with regards to the patient population who would not be able to tolerate lengthy group sessions and to fit with current programming.

### Aim and research questions

The aim of this study was to assess the acceptability and preliminary effects of an adapted Namaste Care program (named Meaningful Moments) which offers an individualized approach with trained volunteers for older adults with mid- to late-stage dementia receiving care at a hospital-based specialized dementia care unit located in a Post-Acute Care hospital. In Ontario, Canada, hospital-based specialized dementia care units in Post-Acute Care hospitals are intended for short-term symptom management care. Patients generally receive care on this unit for 60 days while the healthcare assesses and treats the patient, but many patients stay much longer. They may come from a LTC home or a home in the community and are usually discharged to a LTC home. The research questions regarding the 6-month Meaningful Moments program were: (a) what was the acceptability of the program (e.g., benefits, barriers, facilitators) as perceived by families, staff, volunteers, and charge nurses; (b) what is the preliminary effect of the program on quality of life, neuropsychiatric symptoms, and pain in older adults with mid-to-late stage dementia; and (c) what is the preliminary effect of the program on family carer role stress and the quality of visits for families?

## Methods

### Study design

A mixed methods multiphase design was used [17]. Qualitative description [18] was used for the post-intervention component to explore acceptability of the Meaningful Moments program for families, staff, and volunteers, such as experiences with the intervention, its perceived effects, and barriers and facilitators to implementation. Exploring the acceptability of Meaningful Moments was done to shed light on factors that could have led to effects for patients and families and compare perceived effects to observed effects. A prospective pre-post-test study design was used to evaluate the preliminary effects of Meaningful Moments for patients and families.

### Setting

The setting was a 63-bed hospital-based specialized dementia care unit in a Post-Acute Care hospital for patients with mid- to late-stage dementia. This is a specialized service for adults diagnosed with dementia experiencing responsive behaviours that prevent them from living successfully in another setting such as LTC. The interdisciplinary team was composed of physicians, nurses, therapeutic recreationists, social workers, occupational therapists, physiotherapists, speech-language pathologists, dietitians, charge nurses, clinical managers, as well as a clinical psychologist and clinical nurse specialist. Therapeutic recreationists, also known as recreational therapists, are community college educated and belong to an association. Their role involves assessing patients for appropriate leisure activities and delivering group as well as one-on-one activities. The interdisciplinary team works collaboratively with families and community partners to safely and successfully assess and support the needs of people living with dementia to enable transitions to residential settings such as LTC.

This unit was provided with an opportunity to be one of three sites (the other two sites being LTC settings) for further study of the Namaste Care program within a published study protocol [19]. However, since significant adaptations were ultimately necessary in the specialized dementia care unit, and this changed the delivery of the program, we elected to report separately on findings from the specialized dementia care unit, distinguishing these from findings from the two LTC sites [19–21].

### Meaningful Moments program

We worked with staff at the study site to adapt the intervention to suit their needs and population characteristics. For example, in LTC, Namaste Care is often offered in a group format. However, within this specialized dementia care unit, since the support needs of people living with dementia are still under assessment, many have high levels of agitation, and there is more transition in and out of the community, an individualized approach was better suited to needs. Additionally, instead of having a dedicated room as proposed in Namaste Care, Meaningful Moments programming was delivered naturalistically by volunteers wherever patients were found, including in their rooms or common areas, to avoid causing further distress in patients in transferring to different locations.

Volunteers were sought by posting study information on student volunteer websites and local Alzheimer Society websites. Students in programs related to healthcare (e.g., nursing, therapeutic recreation, health sciences) were also targeted. Four volunteers were recruited for this study to receive training and intense observation. They served as test cases so that a larger pool of future volunteers could receive the same training. Two research assistants were involved in training volunteers. Training consisted of demonstrating how to use Activity Kits for the program to engage with a selected patient, how to include families in sessions, and how to document the interaction using the session checklist. Volunteers were then involved in intensive observation and feedback sessions with the research assistant as they used the activity kits with select patients. Debriefing followed each session.

### Recruitment and participants

The recruitment period lasted from June to September 2018. Patients were required to: (a) be over the age of 65; (b) be English-speaking; (c) have mid- to late-stage dementia; (d) have responsive behaviours of a nature that would not put volunteers at a safety risk (e.g., hitting, kicking, punching, sexually expressive touching); and (e) have a family member or friend who would be able to share the person’s life story and preferences with staff and volunteers. This family member or friend could also be enrolled in the study as a participant or simply provide information to staff to support patients enrolled in the program. Patients were recruited on a rolling basis during this time period, taking into consideration the number of volunteers that were trained. Recruitment was a collaborative process between a research assistant and key administrative contacts using a list of eligible patient-family dyads prepared by the therapeutic recreationists. Using convenience sampling, these administrative contacts would recommend patients who were appropriate for the study based on the eligibility criteria, and actively recruited patients who had family members. Family members were first approached by one of these administrative contacts and if they agreed to be contacted by the research team, a research assistant would initiate a follow up meeting to further explain the study and its goals and to obtain consent. Staff and volunteers with knowledge of the program implementation were recruited to participate in focus groups or interviews.

The patient and family sample size was determined using pilot study considerations [22] and was calculated using Hertzog’s guidelines for determining sample size. The Hertzog’s guidelines suggest 20-25 for participants per site receiving an intervention for a pilot study that intends to demonstrate intervention effectiveness using a one group design [23]. The sample size for staff was considered adequate with the inclusion of staff from diverse perspectives and backgrounds (maximum variation) with regards to age, sex, and number of years of working experience in the current hospital setting. The sample size for each group of respondents (i.e., family carers, staff, charge nurses, and volunteers) was deemed sufficient once no new significant experiences were identified.

### Data collection

Data collection occurred from June 2018 to April 2019. Acceptability was assessed through focus groups with staff from similar disciplines when possible (e.g., charge nurses, therapeutic recreationists, nurses, social workers) and individual interviews with volunteers and family members. With the support of nurses, research assistants collected outcome data at baseline, three months, and six months as per the Namaste Care study protocol. The research assistants received intensive training and standardization testing. Patient quality of life was measured by the Quality of Life in Late-Stage Dementia (QUALID) tool [24]. Other patient outcomes included neuropsychiatric symptoms which were reflective of responsive behaviours (e.g., agitation, anxiety, and depression), measured using the Neuropsychiatric Inventory – Nursing Home Version (NPI-NH) [25]. Patients’ pain was assessed using the Pain Assessment Checklist for Seniors with Limited Ability to Communicate (PACSLAC-II) [26]. Family members who consented to being enrolled in the study as a participant completed surveys with the assistance of research assistants. Family carer role stress (e.g., burden, guilt, conflict, and loss) was measured using the Family Perceptions of Caregiving Role (FPCR) tool [27]. Quality of family visits between people with dementia and their family carers was measured using the Family Visit Scale for Dementia (FAVS-D) [28]. All scales have established reliability and validity and can be used in various settings [25, 26, 28].

### Data analysis

Descriptive analyses of participants’ characteristics were expressed as a mean (standard deviation [SD]) for continuous variables and count (percent) for categorical variables. Age was collected as a categorical variable for family carers, staff, charge nurses, and volunteers. Focus group and individual interview data and notes were analyzed using thematic analysis [29]. Key concepts created from the data were categorized and coded. Two investigators independently analyzed the data to foster credibility and dependability. They reviewed all transcripts and notes independently before coding. Data analysis progressed inductively using a constant comparison approach. We sought similarities and variabilities within the data. Data analysis was conducted in an iterative manner until consensus was reached through discussions with the leads of the team (MY, EC, JS, SK). Several methods were used to improve the credibility of the findings (i.e., data triangulation of data sources and investigators, member checking). With regards to data triangulation of sources, we analyzed transcripts and notes. Our team discussions involved investigators with expertise in dementia, nursing, psychiatry, and quantitative, qualitative, and mixed methods research. In terms of member checking, participants were provided with a verbal summary of key findings following focus groups and interviews to confirm that their perspectives were well captured. See Table 1 for an example of theme development.

**Table 1:** Example of Theme Development

**Data**	**Code**	**Category**	**Theme**
When they [volunteer] would play the music for him I know it would settle him a bit, he liked listening to the music. And when they were reading to him or showing him pictures, he seemed to really look to see what they were. Like he was engaging in it. (Family Carer 1)	Meaningful Moments helped to improve the mood of patients and increased positive responses	Perceived effects of Meaningful Moments for patients	Perceived benefits for patients from Meaningful Moments
I think the other part that’s of value is those moments for our patients that are meaningful become Meaningful Moments for the employee. That’s why we’re all here. Because of the patients that we serve. So if we can get some joy out of seeing their joy, it adds to our day as well. It’s definitely positive reinforcement for employees to see their patients satisfied for a moment. Those are really big wins. (Charge Nurse 3)	Meaningful Moments brought joy for staff in seeing positive responses from patients	Perceived effects of Meaningful moments for staff, volunteers, and families	Staff, volunteers, and families experienced role fulfilment in being able to engage patients^a^
I think you also have to be a little cautious about written communication as well, so if you send out the email, because I speak for myself, but I receive large, large, large volumes of emails and unless it’s actually particularly relevant to my immediate day I likely won’t read it. What is often most helpful is the face-to-face discussions. Certainly, if you want nursing input it really is making sure that you’re accommodating their schedules and when it’s best to communicate with them. (Staff 1)	Information about Meaningful Moments needs to be communicated through in person discussions	Reach of Meaningful Moments	There is a need for a more targeted promotion of the Meaningful Moments program

^a^Please note that the data included in the table only shares the positive impacts of the program for staff as an example and the narrative includes data for staff, families, and volunteers

The outcome results were reported as estimates of model coefficients [parameter estimates, 95% confidence intervals (CIs)] and associated p-values. Statistical significance was set at alpha level of 0.05. All statistical analyses were two-sided and performed using R version 4.2.1. [30]. The generalized estimating equations (GEE) method was used to assess change in the repeated measures outcome data. GEE focuses on the population average change, allows for specification of the correlation structure reflecting variation in the outcomes over time, and does not require the outcome variable to have a particular distribution [e.g., the parametric assumptions were not met by the outcome data thus repeated measures analysis of variance (ANOVA) was inappropriate] [31]. GEE also addresses missing data as it uses all participant data, unlike repeated measures ANOVA which uses only complete cases. Unadjusted models were run for each outcome. Initial model runs assumed an exchangeable correlation structure which is normally recommended as a starting point, but a sensitivity analysis was performed to assess the impact of assuming alternative structures (i.e., independent, unstructured, autoregressive). Since the generalized linear mixed models (GLMM-RI) all ran for the 5 outcomes, which is not always the case with so little data, we reported these results.

### Ethical considerations

Ethics approval was received from the Hamilton Integrated Research Ethics Board (#2865). All participants’ substitute decision-makers received a written introduction to the study and provided informed consent by signing a form written in lay language. Informed consent was obtained from each participant and/or family member in verbal and/or written form.

## Results

### Demographics characteristics

A total of 15 patients participated in the intervention, however demographics were only available for 14 participants. The mean age of patients was 80 years old [(SD) = 9.19]. Most of the patients were male (*n*=10, 66.7%), married (*n*=11), and had been receiving care on the unit for 0-2 years (*n*=13). With regards to family carers, 14 participants provided demographics. Most family carers were over the age of 65 (*n*=10), female (*n*=10), and a spouse to the patient enrolled in the study (*n*=10). See Table 2 for the patient and family carer demographics.

**Table 2:** Patient (*N*=14) and family carer (*N*=14) demographics

**Patient Demographics (*N*=14)**	
**Demographic Variable**	**n (%)**
**Age (years)**	
65-75	4 (28.6)
76-80	4 (28.6)
81-90	5 (35.7)
91 and older	1 (7.1)
**Sex**	
Female	4 (28.6)
Male	10 (71.4)
**Marital Status**	
Married	11 (78.6)
Widowed	1 (7.1)
Divorced	1 (7.1)
Single	1 (7.1)
**Length of Stay in Unit (years)**	
0-2	13 (92.9)
3-5	1 (7.1)
**Family Carer Demographics (*N*=14)**	
**Demographic Variable**	**n (%)**
**Age (years)**	
35-44	1 (7.1)
45-54	1 (7.1)
55-64	2 (14.3)
65 and older	10 (71.4)
**Sex**	
Female	10 (71.4)
Male	4 (28.6)
**Relationship to Patient**	
Spouse	10 (71.4)
Child	4 (28.6)
**Received information about dementia care from current hospital setting**	
Yes	10 (71.4)
No	4 (28.6)
**Level of involvement in planning and delivery of the patient’s care**	
Very involved	6 (42.9)
Somewhat involved	8 (57.1)

The total counts do not add up to 100% due to rounding

A total of 26 staff, charge nurses, and volunteers were included in the study. With regards to staff participants (e.g., nurse, social worker, recreation staff), most were over the age of 45 (*n*=12) and female (*n*=17). All of the charge nurses were between the ages of 45 and 64 and female. There are missing demographic data for the four volunteers who actually delivered the program as once they completed their program of study contact for follow up was lost. These volunteers were students. See Table 3 for the demographics of staff, and charge nurses.

**Table 3:** Staff and charge nurses’ demographics (*n*=22)

**Demographic Variable**	**n (%)**
**Age (years)**	
Staff (e.g., nurse, social worker, recreation staff)	*n*=19
Under 25	1 (5.3)
25-34	2 (10.5)
35-44	3 (15.8)
45-54	7 (36.8)
55-64	5 (26.3)
No response	1 (5.3)
Charge nurses	*n*=3
45-54	1 (33.3)
55-64	2 (66.7)
**Sex**	
Staff (e.g., nurse, social worker, recreation staff)	*n*=19
Female	17 (89.5)
Male	2 (10.5)
Unit mangers	*n*=3
Female	3 (100)
**Number of years working/volunteering in current hospital setting** Mean [standard deviation]	
Staff (e.g., nurse, social worker, recreation staff)	16.1 [12.3]
Charge nurses	29.3 [1.2]

The total counts do not add up to 100% due to rounding. The demographics for the 4 volunteers are missing

### Consultation to develop meaningful moments

To support the adaptation of Namaste Care, consultations with various groups of staff on the unit were initiated to identify perceptions of current programming, including gaps, and how volunteers were involved with patients. Staff included a group of therapeutic recreationists, followed by groups comprised of nurses, social workers, occupational therapists, physiotherapists, charge nurses, as well as a clinical manager, clinical psychologist, and clinical nurse specialist. Consultations with volunteers and families also took place. Staff felt strongly that a gap in programming was one-to-one opportunities for patients who were difficult to engage. Having time to offer such purposeful engagement opportunities was also reported to be an issue, and the potential underutilization of volunteer talents was discussed [21].

During initial consultations with staff [21], it was identified that therapeutic recreationists, along with leaders, were in the early planning stages of a new initiative called Meaningful Moments, that, like Namaste Care, was adapted to supporting person-centred care through positive interactions centred around everyday activities. However, staff felt the structure of a co-planned program could help them to integrate volunteer participation more effectively. Consultations led to a decision that therapeutic recreationists would look to Namaste Care to guide person-centred and multi-sensory inclusions within Meaningful Moments. The novelty of the approach to this program is that volunteers would be delivering it and Namaste Care had not yet been delivered by volunteers in a hospital-based specialized dementia care unit. It has been delivered by hospice volunteers in the UK for people with dementia in a home setting [8].

Therapeutic recreationists assembled “Activity Kits”—a dedicated box for each patient in their rooms that contained a list of suggested activities and supplies for interacting with that particular patient; these activities included walking in the garden or hand massage, and items such as colouring books, sensory balls, games, and CDs. Activities delivered were often passive and provided companionship to meet the needs of the population. Each box was tailored to acknowledge unique abilities and personal interests and would follow the person as they transitioned to LTC. Volunteers would attempt to incorporate at least two or more activities during their sessions and determine which activities captured the interest of patients the most to eventually include these more regularly in future sessions. Plans also included training volunteers to offer moments of enjoyment using the Activity Kits. If volunteers could successfully participate, it would augment what staff alone were able to provide as time was a barrier. The Meaningful Moments program would be the first at the study site to involve staff and families as well as volunteers in initiating meaningful interactions “in the moment” with patients using Activity Kits to support their efforts.

We adapted the typical Namaste Care day program protocol (2 hours in the morning, 2 hours in the afternoon, 7 days a week) [5]. Meaningful Moments involved shorter one-to-one sessions offered when volunteers were available. Volunteers were present only during the weekdays. Sessions would range in length from 15 to 60 minutes depending on the response of the patient and took place in patient rooms or common areas. Consequently, there was no requirement from staff or volunteers to take patients to or from sessions. This structure was meant to decrease the burden of the intervention on the site staff and volunteers and hence make it more feasible to implement as recommended during staff consultations. Meaningful Moments was a new intervention inspired by the Namaste Care program.

Existing volunteers that had been provided by the hospital’s volunteer services department were unable to participate in the study because their volunteer roles had already been negotiated, so external volunteers dedicated to the study were recruited. The volunteers and recreation staff led the actual implementation of the Meaningful Moments program. The rest of the staff and charge nurses were involved by collaborating in delivering the program by using the activity kits and providing advice on the delivery of the program.

### Acceptability

A total of 21 staff participated in post-intervention focus groups and one volunteer and two families completed individual interviews. The mean total number of sessions that each patient received over the course of the 6-month program was 15.07 (SD=12.30). The average duration of sessions was 22.32 minutes (SD=6.94). Themes from the focus groups and interviews were: (a) perceived benefits for patients from Meaningful Moments; (b) staff, volunteers, and families experienced role fulfilment in being able to engage patients; (c) there is a need for a more targeted promotion of the Meaningful Moments program; and (d) individualized programming fosters connections.

#### Perceived benefits for patients from meaningful moments

Families, staff, and volunteers perceived that Meaningful Moments helped to improve the mood of patients and create positive responses in terms of engaging in stimulating activities. Staff also described “going with the flow” and attempting to be intuitive to what the patient needed in the moment—e.g., inviting them into the charting room at night to be around people when they cannot sleep and sharing a story while giving medications. Volunteers were able to engage patients in multiple activities that were enjoyable for patients.

A family carer stated:

When they [volunteer] would play the music for him I know it would settle him a bit, he liked listening to the music. And when they were reading to him or showing him pictures, he seemed to really look to see what they were. Like he was engaging in it. (Family Carer 1)

Staff perceived that the program was most effective when tailored to the life stories of patients. It was important to select activities that resonated with the work that patients used to do so that activities were familiar and soothing.

Some patients reacted really good to them [Activity Kits] because we have one man who used to be a brick layer and if you put that bucket in front of him with all those little blocks, he’s brick laying. So to him it keeps him calm, it keeps him busy, it keeps him quiet and it works for him. So it all depends on what is in the bucket [Activity Kit] and the type of patient. (Staff 8)

A volunteer described how the program appeared to provide patients with a purpose to enjoy meaningful activities and increase their social interactions with others.

They [patients] are more focused [after Meaningful Moments]...I learned that every little moment spent with them makes them confident and independent and just gives them a purpose to enjoy all those meaningful moments again...After Meaningful Moments when I leave, their demeanor and everything changes…So we can do it together and then everyone is looking on and they want to get involved. (Volunteer)

When volunteers were successful in being able to connect with patients through Meaningful Moments, this encouraged others including staff and families to take part in the program as well.

#### Staff, volunteers, and families experienced role fulfilment in being able to engage patients

Charge nurses, staff, volunteers, and families perceived that witnessing positive reactions to the program among patients was rewarding for all. A volunteer noted that an “important thing is showing them that you are also enjoying that moment with them [patient]” (Volunteer). One charge nurse described that Meaningful Moments has value for staff:

I think the other part that’s of value is those moments for our patients that are meaningful become Meaningful Moments for the employee. That’s why we’re all here. Because of the patients that we serve. So if we can get some joy out of seeing their joy, it adds to our day as well. It’s definitely positive reinforcement for employees to see their patients satisfied for a moment. Those are really big wins. (Charge Nurse 3)

This charge nurse alluded to increasing job satisfaction among staff by being able to connect with residents through Meaningful Moments and finding joy in their work.

Staff and volunteers were perceived as having more skills and knowledge through Meaningful Moments which helped them to better engage with patients on an individual level. One therapeutic recreationist reported: “I think a lot of the time it’s in the moment. But sometimes it’s not, it’s about finding that thing that engages that person that you can use again next time” (Therapeutic recreationist 2). Another charge nurse perceived that when staff and volunteers engaged patients outside of their usual task-oriented routines and environments, it helped them to tap into their creativity to connect with this population.

Just seizing the moment and the opportunity to see the patient in a different light, a different setting even. They’re still in this environment but in a different environment in a way... when you see someone in a different light it can help you engage differently as well. (Charge nurse 2)

Staff also acknowledged that although they do document behaviours quite extensively, they rarely document the positive connections they have shared with patients, and discussion in the focus group illuminated this:

…made me think a little about that documentation piece as well, because we’re documenting behaviours…but we’re missing a really big piece…and it made me realize even something small is what worked, and that’s how we literally [in the health record] share it with others. (Staff 9)

A charge nurse similarly acknowledged that there is a need to document positive strategies to address responsive behaviours as much as the behaviour itself: “We should change our way of thinking and chart that [strategies], too. You know, because if we discharge and say, ‘look this and this and this has worked very well’, right” (Charge nurse 1)? The charge nurse was referring to the fact that staff often focus on documenting negative behaviours, and the Meaningful Moments program gave them a chance to recognize and also document positive interactions and behaviours.

The responses to the program were perceived by participants as valuable and families benefited from their interactions, such as witnessing laughter:

When he [husband] would react to things...Especially when he laughs, I just love to see him laugh. You wonder is he laughing, is he really getting it or is something else tickling his fancy. (Family Carer 2).

When families had opportunities to witness positive interactions and behaviours among patients, this increased the quality of family visits and enhanced the wellbeing of families.

#### There is a need for a more targeted promotion of the Meaningful Moments program

To better promote and support the success of the program, a facilitator was perceived by participants as increasing communication between research and unit staff and families using various strategies such as progress updates and face-to-face conversations. Email communication was perceived by staff as being missed at times due to large volumes of emails.

I think you also have to be a little cautious about written communication as well, so if you send out the email, because I speak for myself, but I receive large, large, large volumes of emails and unless it’s actually particularly relevant to my immediate day I likely won’t read it. What is often most helpful is the face-to-face discussions. Certainly, if you want nursing input it really is making sure that you’re accommodating their schedules and when it’s best to communicate with them. (Staff 1)

Participants reported the importance of being flexible in meeting nursing staff schedules when discussing the program as their support was perceived as crucial to the success of the program.

Another facilitator was perceived by participants as keeping families informed and engaging them in the program. Families were found to be receptive to learning that staff and volunteers had successfully engaged with their family members. Staff acknowledged the benefit of passing along information about how someone tried to engage their loved one: “Sometimes I’ll pass that on to family members and they’re just thrilled” (Staff 10). Families were pleased to hear about positive outcomes from staff for a change. Therapeutic recreationists were perceived as helpful in engaging families about the Meaningful Moments.

I would say that our families are educated on the kits...by Therapeutic recreationists who made up the kits. I mean they’re educated on how to engage. They’re given a lot of support and education from like occupational therapists and what have you about where they are at from a communication or whatever perspective. (Charge nurse 3)

Therapeutic recreationists perceived that families also required education in engaging patients which can ultimately lead to greater success when they are aware and prepared to take part in the program.

#### Individualized programming fosters connections

Families, charge nurses, staff, families, and volunteers perceived that patients on the hospital-based specialized dementia care unit required programs offering tailored activities, such as Meaningful Moments. One charge nurse noted that patients on this unit required one-on-one opportunities for engagement.

I think that just comes back to the individually tailored interventions that our patients require. It’s such a specific population that we have on our unit. They require that individual touch so that’s hard to put them in a group program. And that’s exactly the patient that needs Meaningful Moments the most. The ones that cannot tolerate a group or don’t have that ability to engage. They don’t get out to the bigger events. So I think that kind of fit that need in that way. (Charge nurse 2)

Group programs were perceived by participants as difficult for patients experiencing responsive behaviours to tolerate due to multiple triggers for such behaviours related to noise and number of people present. Person-centred care was perceived as important by participants to connect with patients. One therapeutic recreationist described that: “it’s not just about bringing people into a room and making it look like they’re engaged in a sing-along. You have to actually know the person and know what engages them” (Therapeutic recreationist 3). Another participant reported that the success of Meaningful Moments and similar programs were dependent on a thorough assessment “where each and every individual is assessed to the point where we know what their capacities are, what their interests are, what works in terms of changing that behaviour” (Staff 2). Families also reported the importance of programs that offer opportunities for individualized engagement for patients. One family carer described how the Meaningful Moments program brought joy for her, her husband, and a volunteer:

He seems to turn himself off and not want to do something. But with [volunteer] he seemed to listen to the stories. Now, [husband] doesn’t speak in sentences and quite often it’s gibberish that he speaks, but every once in awhile something comes out. And sometimes he would come out with some good, short replies—proper replies which would make [volunteer] laugh, and he’d laugh, and I’d laugh. And it was wonderful. (Family 2)

Witnessing attempts at verbal communication by patients was perceived by families as increasing interactions and connections with volunteers and families.

### Effects

Although there was a trend for slight improvements in QUALID, NPI-NH, and PACSLAC-II scores over time, these results were not statistically significant. See Table 4 for the patient outcome results. There were statistically significant improvements in family carer role stress as FPCR scores decreased over time (parameter estimate of -2.11, 95% CI: -2.79, -1.44; *p* <0.001). With regards to the quality of family carer visits with patients, there were no significant changes in FAVS-D scores over time. Table 5 shows the family carer results.

**Table 4:** Patient outcome results: Parameter estimates (95% Confidence Intervals, *p*-values) for the GLMM-RI Model

**Outcome**	**Mean (SD) at Baseline**	**Mean (SD)** **at 3 Months**	**Mean (SD)** **at 6 Months**	**GLMM-RI Model**		
				**Parameter Estimate**	**95% CI**	***p*-value**
Quality of life of residents (QUALID)	27.07 (7.50) (*n*=15)	27.69 (8.26) (*n*=13)	26.40 (10.95) (*n*=10)	-0.59	(-2.47 to 1.31)	> 0.05
Neuropsychiatric symptoms (NPI-NH)	20.00 (15.61) (*n*=15)	21.08 (12.14) (*n*=13)	20.60 (17.86) (*n*=10)	-0.41	(-3.98 to 3.17)	> 0.05
Pain (PACSLAC-II)	4.27 (4.20) (*n*=15)	2.00 (6.12) (*n*=13)	1.80 (1.99) (*n*=10)	-0.79	(-2.06 to 0.48)	> 0.05

*SD* Standard deviation, *CI* Confidence interval, *QUALID* Quality of Life in Late-Stage Dementia scale (decreased scores reflect improved quality of life; total score ranges from 11 to 55), *NPI-NH* Neuropsychiatric Inventory - Nursing Home scale (decreased scores reflect less behavioural disturbances; total score ranges from 0 to 120 for the first 10 items), *PACSLAC-II* Pain Assessment Checklist for Seniors with Limited Ability to Communicate-II scale (decreased scores reflect less pain; total score ranges from 0 to 60)

**Table 5:** Family outcome results: Parameter estimates (95% Confidence Intervals, *p*-values) for the GLMM-RI Model

**Outcome**	**Mean (SD) at Baseline**	**Mean (SD)** **at 3 Months**	**Mean (SD)** **at 6 Months**	**GLMM-RI Model**		
				**Parameter Estimate**	**95% CI**	***p*-value**
Family carer role stress (FPCR)	269.83 (44.36) (*n*=12)	305.10 (77.36) (*n*=10)	264.25 (61.35) (*n*=4)	-2.11	(-2.79 to -1.44)	< 0.001*
Quality of family visits between persons with dementia and family carers (FAVS-D)	13.38 (6.29) (*n*=13)	10.60 (6.74) (*n*=10)	12.00 (7.75) (*n*=4)	-0.28	(-1.04 to 0.70)	> 0.05

*SD* Standard deviation, *CI* Confidence interval, *FPCR* Family Perceptions of Caregiving Role tool (decreased scores reflect improved perceptions; total score ranges from 81 to 567), *FAVS-D* Family Visit Scale for Dementia (increased scores reflect higher quality of visits; total score ranges from -28 to 28)

^*^denotes a statistically significant result

## Discussion

Key findings included that a volunteer-supported Meaningful Moments program: (a) met the need for tailored interventions for patients with advanced dementia; (b) revealed that volunteers can be trained to provide such interventions; (c) helped staff and volunteers experience rewards in engaging patients with advanced dementia; and (d) decreased stress for families in the family carer role.

Even though Namaste Care was modified, many of the integral components of the program were preserved [5]. Firstly, Meaningful Moments focused on maintaining person-centered care and used a high sensory approach. Each Activity Kit contained specific items that Therapeutic Recreation Staff had successfully used in the past to engage that particular patient. Many of these items were based on the lived experience of these patients. For instance, one patient, a retired mechanic, had several wooden cars in his Activity Kit, which were known to always elicit a positive response from him. Moreover, many Kits contained sensory objects such as light sticks or balls with different tactile sensations. Secondly, the program aimed to capture patients who had advanced dementia and due to their declining abilities were often unable to participate in group programming. While Meaningful Moments did not occur in a dedicated room like Namaste Care would, these sessions most often took place in patient rooms, providing privacy and a familiar environment. If patients were found in the hallway, the volunteer was encouraged to take them to an area that would be quiet and calming in order to have a successful interaction that minimized distractions.

In an implementation study conducted in a special dementia care unit of an LTC home, volunteer-led Montessori based interventions were found to offer meaningful and individually tailored approaches for people with dementia and staff perceived enjoyment and active participation in meaningful activity [32]. Both Meaningful Moments and volunteer-led Montessori based interventions [32] were found to facilitate the successful implementation of the activities by having volunteers deliver them through practical ways such as incorporating activities in the daily flow of routines. In line with our study, Hunter and colleagues found that staff perceived the relationship between volunteers and residents as being the focus of meaningful activities [32]. These findings have implications for practice to ensure that persons living with advanced dementia on hospital-based specialized dementia care units have opportunities for one-on-one connections with volunteers.

The Meaningful Moments program met the need for tailored interventions for patients with advanced dementia on a hospital-based specialized dementia care unit. Although no statistically significant effects were found for patients in the study, staff, volunteers, and family members perceived that patients with advanced dementia experienced benefits including improved mood and more opportunities for social interactions. Gillis and colleagues similarly found that meaningful individual activity leads to decreased depression, aggression, and agitation for residents in LTC [33]. Individually tailored activities can augment the quality of life of persons living with dementia and provide their lives with a purpose and meaning [34]. Family carers of persons with dementia in LTC perceive the importance of personhood in connecting with residents and that positive relationships between staff and residents result by recognizing their history and preferences [35]. This finding supports the need for programs like Meaningful Moments to support staff in moving beyond task-based approaches to care. Person-centred care has been perceived by people with dementia, family members, and staff in LTC as supporting a continuation of self and normalcy by offering meaningful activities, being in a tailored environment, and ensuring flexibility for people with dementia [36]. These findings resonate with our current study findings as offering meaningful activities and upholding the identity of patients were important aspects of Meaningful Moments. In moving towards person-centred approaches charting needs to reflect this as well as recommended by staff in the current study. This practice implication aligns with recent research conducted by Sussman and colleagues who found limited charting evidence that the person-centred preferences noted to be of importance to residents were integrated into care planning [37].

Haunch and colleagues found that staff in LTC still perceived that it was important to connect with residents with advanced dementia and found practical ways to do so such as during personal care [38]. As many responsive behaviours may arise during personal care, Meaningful Moments has the potential to act as a distraction and comforting approach that can be used by staff through activities like music and chatting about things of interest during care. With a common staff perception related to lack of time for staff to meaningfully connect with persons with advanced dementia, there is a need to adapt structured programs such as Namaste Care to suit the culture of hospital-based specialized dementia care units and introduce volunteers as program facilitators. Volunteers can be an important source of support for staff and families as seen in this study as well as a study conducted by Pereira and colleagues [16].

Meaningful Moments was found to support staff and volunteers in experiencing rewards in engaging patients with advanced dementia through a sense of role fulfilment. Staff and volunteers felt reciprocal joy when they were able to connect with patients through individualized activities. Cheloni and Tinker explored the motivations of nurses and healthcare assistants in working with older adults in a hospital setting and revealed that these staff were motivated to be in these roles based on inner rewards such as a sense of fulfilment and accomplishment [39]. When staff are able to recognize that their engagement is making a difference in the lives of persons living with dementia through small cues such as a smile, this offers positive reinforcement for staff to continue to find ways to connect with this population [38] and can contribute to staff retention. These findings reveal the need to ensure that staff and volunteers continue to persevere in their efforts to meaningfully engaging patients with dementia and be able to recognize when their efforts have made an impact in the quality of life of patients. Prospective volunteers should be informed of the rewards in working with persons with dementia to support their continued efforts and for volunteer retention.

Despite positive perceptions of staff, family and volunteers regarding the Meaningful Moments program for patients, we did not find any statistically significant findings. These findings similarly align with a study evaluating Namaste Care in LTC, however in that study there was an improvement in resident neuropsychiatric symptoms at the 3-month time point only [20]. With regards to the effects of the Meaningful Moments for family carers, we found a statistically significant improvement in family carer role stress. This finding aligns with the same study evaluating Namaste Care in LTC [20] and reveals the importance of measuring effects of interventions for both patients and families. Despite the program sessions being offered far fewer and shorter than the original Namaste Care protocol and only 20 minutes at a time, families still benefited from the program. With more frequent and longer sessions, this may improve effectiveness of the program. Similar to Meaningful Moments, Montessori-based interventions have been found to lead to greater satisfaction with visits for family carers of persons with dementia in LTC [40]. Spousal carers have been found to require support that focuses on the positive aspects of caregiving the reduce the negative impacts such as stress and burden [ [41]. The Meaningful Moments program also has the potential to facilitate the transition from the hospital-based specialized dementia care unit to LTC by decreasing responsive behaviours.

### Strengths and limitations

The strengths of this study included the use of both quantitative and qualitative methods to explore acceptability and preliminary effects of Meaningful Moments and the inclusion of families, volunteers, and staff in the sample. A key strength of this study is that staff, especially therapeutic recreationists, co-designed the Meaningful Moments program, which increases the relevance of the program and can ensure its sustainability in sites where it is implemented [42]. The major limitation of this study was that the Meaningful Moments session frequency and duration were well below the levels of the Namaste Care protocol that would be needed to see a sustained effect. It was also difficult to recruit more volunteers for the role of program facilitator with reliance due training capacity at the site which may have been part of the reason why so few sessions were delivered. As Meaningful Moments was tailored for each patient, different activities may have been delivered by volunteers for each patient and the results reflect all activities grouped together. It was difficult to evaluate whether different activities had greater effectiveness than others as these are all dependent on the unique needs, interests, and contexts of people living with dementia. Other limitations of this study were the small sample size of families and patients which did not allow for an adjusted model that included covariates to measure effects of the intervention and lack of a diverse sample with regards to race and ethnicity, which may not accurately reflect the makeup of the Canadian population [43]. Future research should consist of a pragmatic randomized controlled trial with a larger, more representative sample to measure the effects of the Meaningful Moments program.

## Conclusions

In this study, we explored the acceptability and preliminary effects of involving trained volunteers in the Meaningful Moments program for older adults with dementia receiving care at a hospital-based specialized dementia care unit located in a Post-Acute Care hospital. Volunteers were found to augment the efforts of staff to meaningfully engage patients using a one-on-one approach. Findings revealed the importance of offering individually tailored interventions for older adults with advanced dementia through practical strategies based on knowledge of the person that can offer benefit to patients. These activities should be provided as a way for staff, families, and volunteers to foster connections and build relationships with patients.

## Data Availability

The data for this research consists of questionnaires and interviews and focus group transcriptions and notes. Raw data cannot be publicly released due to the risk of compromising participant confidentiality. The datasets used and/or analysed during the current study available from the corresponding author on reasonable request.

## Abbreviations

HCP: Healthcare provider
LTC: Long-term care
SD: Standard deviation
